# Uses of Andersen health services utilization framework to determine healthcare utilization for mental health among migrants—a scoping review

**DOI:** 10.3389/fpubh.2023.1284784

**Published:** 2023-12-12

**Authors:** Ewa Zuzanna Krzyż, Oscar Fidel Antunez Martinez, Hung-Ru Lin

**Affiliations:** ^1^PhD Program, School of Nursing, National Taipei University of Nursing and Health Sciences, Taipei, Taiwan; ^2^School of Nursing, National Taipei University of Nursing and Health Sciences, Taipei, Taiwan

**Keywords:** migrants, mental healthcare utilization, Andersen health system utilization framework, scoping review, public health

## Abstract

**Background:**

Migration is a worldwide occurrence that carries significant implications for healthcare systems, and it entails challenges to mental healthcare. The Andersen Behavioral Model is widely used by researchers to determine healthcare service utilization among many populations, including migrants. Our study aimed to explore the ways of using the Andersen Health System Utilization Framework in the literature to discover the utilization of mental healthcare by migrants.

**Methods:**

This scoping review was based on Arksey and O’Malley’s framework. A comprehensive search was performed across five electronic databases.

**Results:**

A total of 12 articles from January 1992 to July 2023 identified various versions of the Andersen Behavioral Model to provide an overview of mental health services utilization among migrants. The analysis identified four significant trends in the literature. First, there is a predominant focus on individual characteristics over contextual factors. Second, researchers tend to integrate multiple versions of the Andersen Behavioral Model, and the most is the version from 1995. Third, additional factors specific to migrant populations are incorporated into the model, but the categorization is sometimes unclear. Finally, the majority of studies have used a quantitative approach and are based in North America, suggesting a focus on the significance of mental health in migrant communities in that context.

**Conclusion:**

In summary, our scoping review calls for further research using the Andersen Behavioral Model to study mental healthcare utilization among migrants. Notable findings include the adaptation of the model to migrant populations, a focus on individual characteristics, a need for more diverse research methods, and the proposal of a new conceptual model to guide research and policy development in this field.

## Introduction

Migration is a worldwide occurrence that carries significant implications for healthcare systems (1). According to the United Nations Global Migration statistics, in the year 2020, there were 281 million international migrants, which accounts for 3.6% of the world’s population (2). Due to the intricate nature of migration, it frequently entails various challenges and hazards that contribute to stress, burden, and risk factors (3, 4). These may include inadequate access to healthcare services and the separation of families and children from their loved ones and other relatives (3, 5). Additionally, migrants may face difficulties such as homelessness, insufficient food and water, xenophobic attacks, limited educational opportunities, perceived and actual discrimination, and a heightened vulnerability to death and injuries (6).

Therefore, according to a comprehensive analysis conducted by Henssler et al. (7), it was found that both first- and second-generation migrants and refugees exhibit higher prevalence rates of schizophrenia and related psychoses than native populations. In a systematic review conducted by Morina et al. (8), it was observed that among refugees and internally displaced persons following forced displacement, the highest prevalence rates of psychiatric disorders were found for post-traumatic stress disorder (ranging from 3 to 88%), depression (ranging from 5 to 80%), and anxiety disorders (ranging from 1 to 81%) (8). Additionally, it has been shown that compared to the general population, migrants are less likely to seek out care for mental health conditions due to barriers (9, 10).

Numerous factors, including cultural, linguistic, socioeconomic, and legal issues, impact the patterns of mental healthcare utilization among immigrants (11). Furthermore, it is acknowledged that demographic attributes such as age, gender, educational attainment, and overall health status are factors that may influence the utilization of mental health services (12–15). However, research examining these factors and the utilization of mental healthcare for immigrants remains inconsistent and faces methodological limitations (16). In our scoping review, we aim to systematize existing knowledge on this topic and provide evidence-based recommendations for healthcare providers, researchers, and policymakers. Moreover, it has been suggested that a literature review serves as the foundation for conducting high-quality research in medical fields, enabling researchers to maximize the relevance, originality, generalizability, and impact of their work while ensuring that professional standards are met (17).

Healthcare utilization refers to individuals actively engaging with the healthcare system to prevent and treat health issues, enhance overall health and well-being, or gather information about their health condition and future outlook (18). Various models have been developed across diverse fields of study to analyze and predict the intentions and behaviors of individuals when utilizing healthcare services (19). The Behavioral Model of Health Services Use (BMHSU) is extensively referenced in health services research, particularly concerning healthcare service utilization (20). The model primarily emphasizes three fundamental factors that contribute to explaining healthcare utilization: predisposing factors (such as age and education), enabling factors (such as income and availability of healthcare facilities), and need factors (such as overall health status) (21).

In the present day, various adaptations of the model are available and employed in health services research, customized for different settings or specific target groups. The original version of the Andersen Behavioral Model was formulated in the 1960s, proposing that individuals’ utilization of health services is determined by three key factors: their inclination or predisposition to use services, the enabling (resources) or hindering factors that facilitate or impede use, and their actual need for care (22).

**Figure 1:**
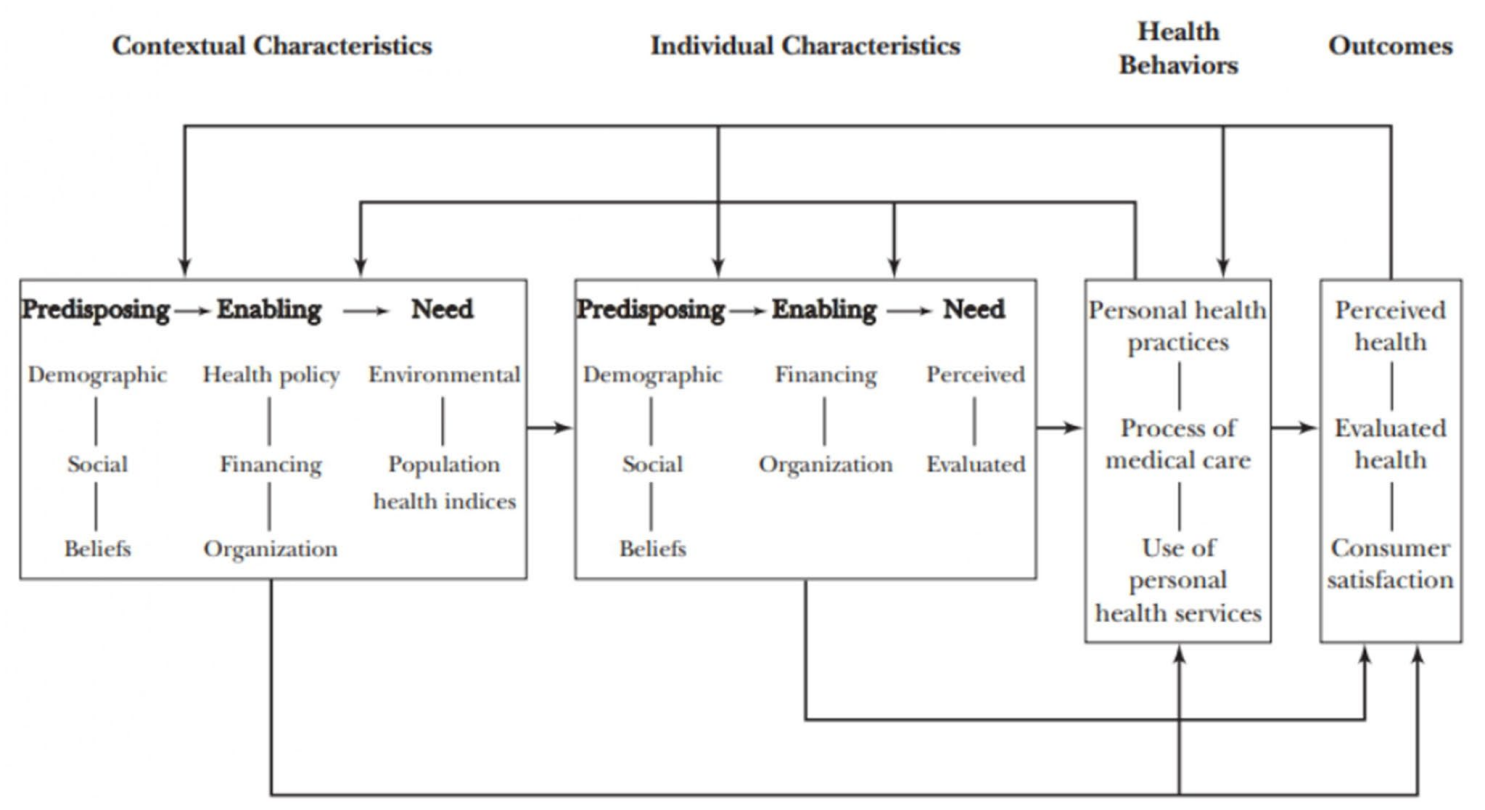
Andersen Behavioral Model of health service utilization (26).

In recent years, Andersen’s original behavioral model has undergone continuous development, with a focus on incorporating various factors. For instance, in the 1970s, there was an emphasis on “consumer satisfaction” (23, 24), while the 1980s saw the inclusion of “health status,’’ “personal health practice,’’ and the “external environment” (22, 25). In 1995, Andersen himself reviewed and updated the model, introducing feedback loops to consider how treatment outcomes impact health behavior. In the 2000s, additional elements such as “contextual and individual characteristics” were incorporated into the model (21). Figure 1 illustrates the selected fundamental components and their relationships within the most current version of the Andersen Behavioral Model (26).

Several systematic reviews have already explored the application of the Andersen Behavioral Model and its various versions. These reviews specifically focus on particular settings (27), diseases (28), or general healthcare (29). Additionally, there is a systematic review that presents qualitative uses of the Andersen Behavioral Model (30). One study from 2016 provided a theoretical framework for explaining immigrant health service utilization, focusing on health in its general meaning (31). However, Yang and Hwang (31) concentrated on developing a framework rather than conducting a comprehensive literature review. Unlike broader systematic reviews that cover general healthcare or specific diseases, a scoping review dedicated to mental healthcare utilization among migrants can provide in-depth insights into a specific and often underserved area of healthcare, especially since mental health significantly differs from physical health. Moreover, our scoping review aims to examine how multiple factors interact within the context of migrants’ mental health. Our research does not focus specifically on one methodological approach; rather, we seek to integrate both qualitative and quantitative data to allow for a more comprehensive understanding of the topic. Thus, the overall purpose of this article is to explore the ways of using the Andersen Health System Utilization Framework in the literature with the purpose of discovering the utilization of mental healthcare by migrants.

## Materials and methods

To achieve the objectives, a scoping review was undertaken. This methodology is considered a rigorous and valuable approach for identifying research gaps and offering directions for future studies in the field (32, 33). The development of our scoping review was guided by the recommendations for conducting a scoping review following five steps of Arksey and O’Malley (32): (1) defining the research question, (2) identifying relevant studies, (3) defining the study selection, (4) charting the data, and (5) collecting, summarizing, and reporting the result.

### Defining the research question

After identifying the specific area to be addressed in this review, we formulated a comprehensive primary research question with a broad scope: what is currently known within the literature about factors specified in the Andersen Health System Utilization Framework affecting mental healthcare service utilization for individuals with a migration background?

Therefore, to facilitate the analysis of the findings, the study included the following additional sub-questions: (1) What are the uses of different versions of the Andersen Health System Utilization Framework among migrants? (2) Which factors specified in the Andersen Health System Utilization Framework are most commonly identified when examining mental healthcare service utilization among migrants? (3) What conceptual model specifically addresses mental healthcare utilization among immigrant populations?

### Identifying relevant studies

Based on a preliminary search, the authors implemented the following steps:

1. Key search terms and their MeSH terms—for a comprehensive search, “mental health” was deliberately excluded as one of the primary keywords. This strategy aimed to prevent situations where authors in the articles did not clearly distinguish between mental and physical health. We used keywords such as migrants/immigrants, Andersen Health System Utilization Framework, and healthcare utilization. Additionally, MeSH terms and synonyms of the mentioned keywords were employed to capture a broader range of relevant literature, providing a more inclusive understanding of the topic.
2. Choice of database—PubMed, CINAHL, APA PsycArticles, MEDLINE, and Web of Sciences. We selected these databases because they prioritize high-quality, peer-reviewed content across multiple disciplines, including healthcare, psychology, social sciences, and public health, aligning with the scope of our research.
3. Inclusion criteria: the review focused on empirical studies on health service utilization published in English from January 1992 to March 2023, addressing our research question. It was essential that the studies utilized the Andersen Behavioral Model to explore factors associated with mental healthcare utilization. The review specifically focused on adult migrants or immigrants (aged 18 years and older).

### Study selection

**Figure 2:**
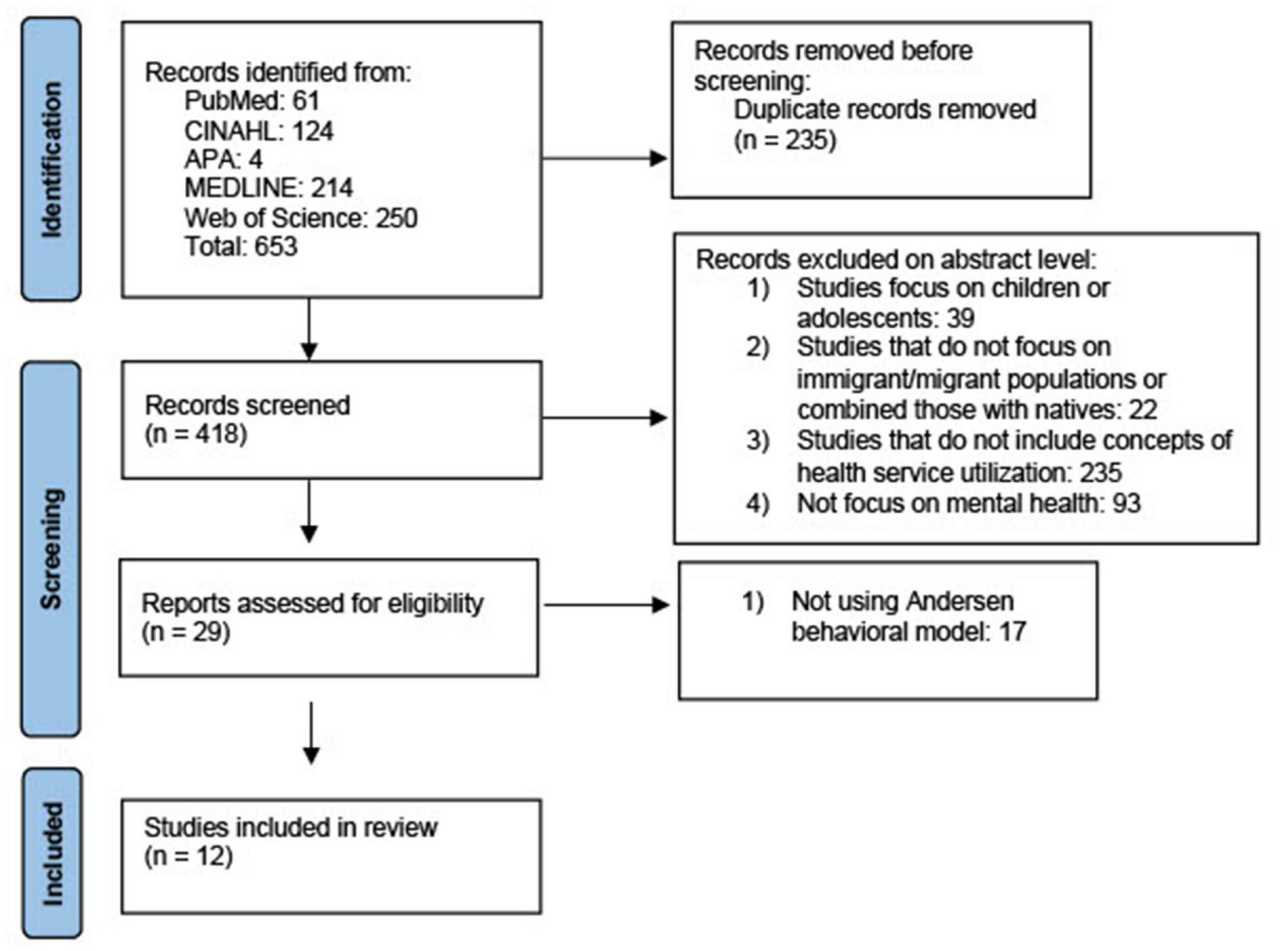
PRISMA diagram for screening process (34).

The authors conducted the search process using the citation manager EndNote®v.20.2.1, created by Thomson ISI Researchsoft and sold by Camelot UK Bidco Limited. In cases where abstracts lacked sufficient information, complete articles were thoroughly assessed to determine their relevance. The detailed screening process is illustrated in Figure 2, following the steps outlined in the PRISMA diagram, a validated tool for Reporting Items for Systematic Reviews and Meta-Analyses (34).

After conducting an initial search, the authors identified 653 articles. Upon removing duplicate and triplicate articles, 418 studies were deemed potentially relevant to the review. The authors conducted a title and abstract screening process to identify studies with potential relevance, dividing the studies equally among the authors. As the decision was made to focus exclusively on the adult population, the authors excluded studies that included children and adolescents from further consideration. Correspondingly, studies irrelevant to the study populations or the specific concepts under review were also excluded.

During this stage, the review excluded studies that did not primarily focus on mental health. Consequently, the authors decided to narrow their focus exclusively to articles that specifically addressed migrant or immigrant populations, excluding any studies that compared this group with native populations. Because the migrant population faces challenges that are different from those of natives in seeking mental healthcare, which may influence their decision-making process, we specifically aimed to focus on this population to better discover and understand their factors. This decision was made with the purpose of gaining a more comprehensive understanding of the unique experiences faced by migrants or immigrants. The goal was to create outcomes that provide more applicable insights for interventions and policies tailored to migrant populations.

After the initial screening, a total of 29 articles appeared to be potentially relevant to the review. These articles were subsequently retrieved in full-text format for further screening and evaluation. Using the same inclusion criteria, the authors proceeded with screening the full-text articles. To ensure the reliability and objectivity of the study, the researchers independently reviewed each full text, and any disagreements were discussed until a consensus was reached. This screening engaged the reviewers. The purpose of the second screening was to exclude academic articles that did not use the Andersen Behavioral Model. Finally, after applying the search criteria and conducting a thorough review, the reviewers determined that 12 articles met the criteria and were considered suitable for inclusion in the final review. These academic articles were then subjected to data extraction for further analysis.

### Charting the data

Following the recommendation of Arksey and O’Malley (32), the 12 selected studies were systematically reviewed and organized. Relevant information was gathered and structured into Excel tables with categories such as author, year of publication, country of publication, participants’ characteristics, study design, method of data collection, method of data analysis, and result. This approach was employed to enhance the reliability of the data extraction process. Next, the review classified and recorded in an electronic spreadsheet designed for data extraction purposes the variables identified by the Andersen Model in the studies. First, we collected information about versions of the Andersen Model used in each study. Next, the key components of the template included domains based on the Andersen Model (26): contextual variables, individual characteristics, health behavior variables, outcomes, and sub-domains: (predisposing, enabling, and need factors). Each of the selected studies was thoroughly reviewed and coded based on our coding template. We organized and entered data from the included studies into an electronic spreadsheet, with a particular focus on categorizing specific variables that were measured. Afterward, both authors shared equal responsibility for the data extraction and review. Adopting this approach ensured the validity of the extraction procedure in the scoping review.

### Summarizing and reporting the results

We used Microsoft Excel to compute descriptive statistics to provide a descriptive overview. Next, we summarized the uses of the Andersen Model and its factors among the selected studies. Whenever a new factor emerged, the categorization system was extended. Subsequently, a summary was generated for each category.

By implementing this method, we were able to assess the included studies in a standardized manner, facilitating the categorization, classification, and comparison based on shared characteristics, variations, and areas of research gaps. Subsequently, the authors used a table to present the findings.

## Results

### Overview of characteristics of included literature

Our comprehensive evaluation and examination covered a total of 12 articles (35–46), and an overview of the key features and attributes of these articles is available in Table 1. Most of the research that fits into the inclusions and exclusions criteria was quantitative (*n* = 11), and one study used a mixed-methods approach. However, for this study, we chose to include only the quantitative part to maintain consistency in analyzing the results for our scoping review. The majority of the research studies were conducted in or originated from the United States (*n* = 11). Additionally, the review included one study from Canada. It is worth noting that the reviewed studies spanned over 29 years, indicating a wide range of publication dates (1992–2021).

**Table 1:** Characteristics of reviewed studies.

Author	Year	Country	Participants, Sample size	Study design	Data collection	Result
Baek et al. (35)	2020	United States	Korean Americans (*n* = 243)	Quantitative	Survey	Significant factors: predisposing and enabling (individual characteristics), needs factors
Chao et al. (36)	2020	United States	Older Chinese immigrants (*n* = 130)	Mixed-method	Survey	Significant factors: predisposing and enabling (individual characteristics), needs factors
Fenta et al. (37)	2006	Canada	Ethiopian immigrants and refugees (*n* = 342)	Quantitative	Survey	Significant factors: predisposing and needs factors (individual characteristics)
Hochhausen et al. (38)	2011	United States	Latina immigrants (*n* = 262)	Quantitative	Databases	Significant factors: predisposing, enabling, and needs (individual characteristics)
Kim et al. (39)	2010	United States	Latino and Asian immigrants (*n* = 501)	Quantitative	Databases	Significant factors: predisposing and needs (individual characteristics)
Lee and Held (40)	2015	United States	Latino immigrants (*n* = 2,533)	Quantitative	Databases	Significant factors: predisposing, enabling, and needs (individual characteristics)
Lee and Jang (41)	2016	United States	Korean immigrants (*n* = 205)	Quantitative	Survey	Significant factors: predisposing, enabling, and needs (individual characteristics)
Lee et al. (42)	2014	United States	Latino and Asian immigrants (*n* = 1,444)	Quantitative	Survey	Significant factors: predisposing, enabling, and needs (individual characteristics)
Park et al. (43)	2013	United States	Korean Americans (*n* = 363)	Quantitative	Survey	Significant factors: predisposing, enabling, and needs (individual characteristics)
Park et al. (44)	2018	United States	Korean Americans (*n* = 420)	Quantitative	Survey	Significant factors: predisposing, enabling, and needs (individual characteristics)
Portes et al. (45)	1992	United States	Mariel Cubans and Haitian refugees (*n* = 952)	Quantitative	Survey	Significant factors: predisposing, enabling, and needs (individual characteristics) and contextual characteristics
Saasa et al. (46)	2021	United States	African immigrants (*n* = 323)	Quantitative	Survey	Significant factors: enabling and needs factors (individual characteristics)

The source data varied and included primary data collection (*n* = 9) or large national administrative databases (*n* = 3). Most studies focused on Korean Americans (*n* = 4) and Latino immigrants, often combined with other immigrant populations (*n* = 4). Two studies focused on African immigrants. One study focused on Mariel Cubans and Haitian refugees, and another on Chinese immigrants. The sample size varied from 130 (36) to 2,533 (40). All studies conducted simple statistical analyses at the univariate and bivariate levels. Five of the selected studies conducted additional multilevel analyses (35–37, 39, 46). Our selected studies found a significant relationship between mental healthcare utilization (formal and/or informal) influenced by needs factors (100% of the studies), predisposing factors (91.6% of the studies), and enabling factors (83.3% of the studies). Moreover, the contextual characteristic has also been found to be an important one when exploring mental healthcare utilization (45).

### Applied model versions

Various versions of the Andersen Behavioral Model were discovered and employed in the studies under review. Our findings indicate that:

- The most frequently used version was from 1995 (22), employed by five of the selected studies to explore mental healthcare utilization among migrants (35, 38–40, 42).
- Two other studies (41, 43) used the original Behavioral Model of Families’ Use of Health Services version from 1968 (24).
- The remaining selected studies combined more than one version of the Andersen Behavioral Model.

Moreover:

- Several studies employed variations of the Andersen Behavioral Model by combining different versions of the framework. For instance:
- Park et al. (44) integrated the 1995 version by Andersen (22) with the 2005 version developed by Andersen and Newman (47).
- Saasa et al. (46) combined the 1995 version (22) by Andersen with the 1968 version (20).
- Fenta et al. (37) merged the 1995 version (22) with a version where Andersen collaborated with Newman in 1973 (24).
- Chao et al. (36) utilized three different versions of the Andersen Behavioral Model, namely, the 1995 version (22) and the versions in which Andersen collaborated with Aday (23) and Newman (47).
- Portes et al. (45) incorporated the version by Aday (23) and Newman (24) while also using the 1968 version by Andersen (20).

### The Andersen Behavioral Model: factor distribution

Among the 12 studies that explored mental health service utilization among migrants using the Andersen Behavioral Model, the focus was primarily placed on individual characteristics and health behaviors rather than contextual characteristics and health outcomes. While the studies included various factors from the Andersen Behavioral Model and introduced specific factors for the migrant population, there were still several factors from the original theory that were not considered in any of the 12 studies. Table 2 provides a general overview of the distribution of factors among the selected studies. Meanwhile, Table 3 presents detailed information on specific factors distribution among the articles.

1 *Contextual variables:* Only a single study explicitly addressed contextual variables by representing them through country of birth and distinguishing between rural and urban areas and co-ethnic communities (45). This study also suggested considering the patterns that migrants used in their country of origin when seeking mental health assistance as these patterns might significantly influence their mental healthcare seeking in the new country (45).
2 *Individual characteristics:* Predisposing factors were found to be the most common group of factors among individual characteristics.

- Age (100%), gender (83.3%), marital status (75%), and immigration-related factors (66.6%) were frequently examined by researchers. Three other studies examined racial/ethnic differences (39, 40, 42), which can be considered also as a predisposing factor.
- The review revealed that researchers frequently incorporated additional factors, particularly within the enabling group. Then, the review distinguished and labeled these factors as “determinants of mental health for migrants,” which encompassed immigration-related factors, health/illness-related factors, education, resiliency, and perceived need for help. The other general factors that have been examined as enabling were those related to financing (health insurance, income/poverty, and employment status), organization (affordability and accessibility of mental health services), and social support.
- The need factors in our review were categorized as perceived (self-rated mental health, perceived need for help, postmigration stressful life events, and work productivity loss) and evaluated (mental illness-related factors, number of somatic disorders, and chronic medical condition). Among those, the group of evaluated factors was more frequently examined compared to the perceived ones.
- While the Andersen Behavioral Model was not originally developed for the migrant population, this scoping review focused on articles related to migrants and, as a result, identified numerous additional factors specific to this population within the selected studies. These factors included acculturation (35, 41, 43, 44, 46), English proficiency (36, 37, 39, 43, 45), years spent abroad or in the new community (36–39, 41, 43), generational status (35, 46), having children in the home country (38), and age at emigration (37).
- Researchers did not clearly distinguish between predisposing and enabling factors when considering these migrant-related factors. Additionally, two studies presented them as an independent group of factors (40, 42). Only one factor, post-migration life events, was identified as specifically related to immigration among their need factors.

3 Health behavior variables: All the studies included in the analysis identified their primary measure as the utilization of mental health services, falling under the category of “Use of personal health practices” according to the Andersen Behavioral Model (26). Personal health practices were observed in only four of the studies, primarily as a component of mental health service utilization.
  Furthermore, one study specifically focused on the use of antidepressants, which can also be considered a personal health practice. None of the studies mentioned smoking, alcohol consumption, diet, or exercise as indicators of health behavior. Additionally, none of the selected studies explored the relationship between patients and healthcare providers during the care process.
4 *Health outcomes:* The selected studies did not investigate health outcomes such as perceived health, evaluated health, or consumer satisfaction. Instead, all the studies focused on utilizing the Andersen Behavioral Model to examine health behavior variables as their primary outcomes.

**Table 2:** General summary of the review studies based on the Andersen Behavioral Model.

Author	Contextual variables	Individual variables			Health behavior variables	Health outcomes
		Predisposing	Enabling	Need		
Baek et al. (35)	No	Yes	Yes	Yes	Yes	No
Chao et al. (36)	No	Yes	Yes	Yes	Yes	No
Fenta et al. (37)	No	Yes	Yes	Yes	Yes	No
Hochhausen et al. (38)	No	Yes	Yes	Yes	Yes	No
Kim et al. (39)	No	Yes	Yes	Yes	Yes	No
Lee and Held (40)	No	Yes	Yes	Yes	Yes	No
Lee and Jang (41)	No	Yes	Yes	Yes	Yes	No
Lee et al. (42)	No	Yes	Yes	Yes	Yes	No
Park et al. (43)	No	Yes	Yes	Yes	Yes	No
Park et al. (44)	No	Yes	Yes	Yes	Yes	No
Portes et al. (45)	Yes	Yes	Yes	Yes	Yes	No
Saasa et al. (46)	No	Yes	Yes	Yes	Yes	No

**Table 3:** Distribution of factors among selected studies.

Factors:	Total *N* = 12	*N* = (%)
*Contextual characteristics:*		
1. Predisposing factors	/	/
2. Enabling factors	/	/
3. Needs factors	/	/
Environmental		
• Country of birth (45)	1	8.3%
• Co-ethnic Community (45)	1	8.3%
Population health indices	/	/
*Individual characteristics:*		
1. Predisposing factors:		
Demographic		
• Age (35–46)	12	100%
• Gender (35–45)	10	83.3%
• Marital status^*^ (37, 39–46)	9	75%
• Immigration-related factors^*^ (35–38, 41, 43–46)	8	66.6%
• Racial/ethnic differences (39, 40, 42)	1	8.3%
• Religion^*^ (43)	3	25%
Genetic	/	/
Social		
• Education (36, 38–45)	9	75%
• Family-related factors^*^ (38)	1	8.3%
Health beliefs	/	/
2. Enabling factors:		
Financing		
• Health insurance (35, 38, 40–43, 46)	7	58.3%
• Income/poverty status (35, 42, 43)	3	25%
• Employment status (37)	1	8.30%
Organization		
• Affordability and accessibility of treatment/health services (38, 45)	2	16.6%
Social support^*^		
• Social support/network (35–37, 40, 42)	5	41.6%
Determinants of mental health for migrants^*^		
• Immigration-related factors^*^ (35–37, 39, 41, 43, 46)	7	58.3%
• Health/illness related factors^*^ (36, 41, 44)	3	25%
• Education^*^ (35, 37, 46)	3	25%
• Resiliency^*^ (35)	1	8.3%
• Perceived need for help^*^ (43)	1	8.3%
3. Needs factors:		
Perceived		
• Self-rated mental health (39, 44)	2	16.6%
• Perceived need for help (36)	1	8.3%
• Postmigration stressful life events^*^ (37)	1	8.3%
• Work productivity loss^*^ (46)	1	8.3%
Evaluated		
• Mental illness-related factors (35–46)	12	100%
• Number of somatic disorders (37)	1	8.3%
• Chronic medical condition (46)	1	8.3%
*Health behavior variables*		
1. Personal health practices		
• Complementary and alternative medicine (37, 42)	1	8.3%
• Personal coping strategies (35)	1	8.3%
• Taking antidepressant^*^ (44)	1	8.3%
2. The process of medical care	/	/
3. Uses of personal health practices		
• Health services use (35–46)	12	100%

^*^These factors were developed along the data material. Immigration-related factors include acculturation, English proficiency, length of stay abroad, generational status, having children in home country, and age at emigration.

### Trends in the selected studies

Through our scoping analyses, we identified four significant patterns and trends that are currently prevalent in the literature.

Firstly, among the studies analyzed, only one specifically examined the contextual components, while the remaining studies primarily focused on individual characteristics.

Secondly, researchers tended to integrate multiple existing versions of the Andersen Behavioral Model when examining the seeking behavior of mental healthcare among migrants. This trend was evident in five of the studies in our review, compromising 41% of all the studies.

Thirdly, there was a pattern of including additional factors specifically designed for the migrant population in the Andersen Behavioral Model. However, these factors were often mixed between enabling and predisposing variables without a clear explanation. Moreover, two studies (40, 42) added immigrant factors as another group of factors and did not directly include them in the Andersen Behavioral Model.

In addition, the majority of studies investigating the utilization of mental healthcare services among migrant populations employed a quantitative research approach and originated from the United States. This observation implies that the United States may have a greater awareness of the significance of mental health in migrant communities.

These four trends might suggest the need for further development of the Andersen Behavioral Model, specifically tailored to examine mental health patterns among migrants. This improvement is crucial to enhance health outcomes for this population and gain a deeper understanding of their healthcare utilization.

## Discussion

This scoping review presents an up-to-date summary of how the Andersen Behavioral Model has been employed among migrant populations to identify their patterns of mental health services utilization. By employing the model, we were able to identify the strengths and limitations present in the existing literature and build a conceptual framework that can be used for further reference when examining this concept.

First of all, the scoping review identified 12 studies, all of which were conducted in North America and mostly utilized a quantitative study design. A similar pattern emerged in a scoping review of the Andersen Behavioral Model and its uses in healthcare service utilization for the general population (30), where 70% of the studies were from North America, with 89% of them employing a quantitative study design. On the one hand, the consistency of the findings within the selected country strengthens their validity. In addition, our studies, even mostly from North America, combined a wide range of immigrant populations, including Korean Americans, Latino immigrants, African immigrants, Mariel Cubans, Haitian refugees, and Chinese immigrants. This diversity is novel and reflects the multifaceted nature of migrant healthcare utilization. On the other hand, this limitation means that the findings may not directly apply to healthcare systems in other countries when studying the utilization of services by migrants, and thus, this can limit the generalizability of the results to other geographic locations or populations. Indeed, different regions may have unique environmental, cultural, economic, or social factors that influence the outcomes of studies. In addition, the policy recommendations derived from these findings may not be relevant or applicable to areas outside the scope. Policymakers may need to consider the local context when applying the review’s conclusions to their specific jurisdiction. They should consider adapting the Andersen Behavioral Model to their specific context and population. This might involve adding or emphasizing certain factors that are more relevant to the healthcare-seeking behavior of migrants in their region. Furthermore, this limitation suggests the need for future studies to explore similar topics in different countries. These findings indicate that further research exploring this topic in diverse countries is necessary to provide a more comprehensive overview of this concept, employing a qualitative or mixed-methods approach to better understand participants’ perspectives and obtaining more reliable results by keeping the balance between the strengths and weaknesses of quantitative and qualitative study designs.

Furthermore, our research revealed that the most utilized framework for examining mental healthcare service utilization among migrants was the 1995 version of the Andersen Behavioral Model. This finding aligns with the results presented by Babitsch et al. (29), who also identified the Andersen Behavioral Model from 1995 as the most frequently employed. Nevertheless, unlike our review, their research did not identify a trend of combining multiple existing versions of the Andersen Behavioral Model when investigating healthcare service utilization. This suggests that a more comprehensive approach may be necessary when addressing migrants’ mental healthcare-seeking behavior. Additionally, Babitsch et al. (29) found that most of the reviewed studies utilized secondary data analyses, whereas in our scoping review, only three studies employed secondary data (38–40), and the use of primary data was dominant.

Thirdly, there was a consistent pattern where researchers often overlooked contextual characteristics and predominantly focused on individual factors, particularly enabling and predisposing factors. Similar patterns were identified in a scoping review of the Andersen Behavioral Model for secondary complications of spinal injury (27) and a scoping review for the general population (30). However, unlike our review, both reviews still identified studies that explored health outcomes. This suggests that there are a limited number of studies focusing specifically on mental health service utilization among migrants, with a lack of an appropriate framework tailored to this population. Additionally, the existing theoretical framework proposed by Yang and Hwang (31) does also not address this group of factors. This might suggest that in understanding migrants’ healthcare-seeking behavior, factors related to ‘health outcomes’ do not play a significant role.

**Figure 3:**
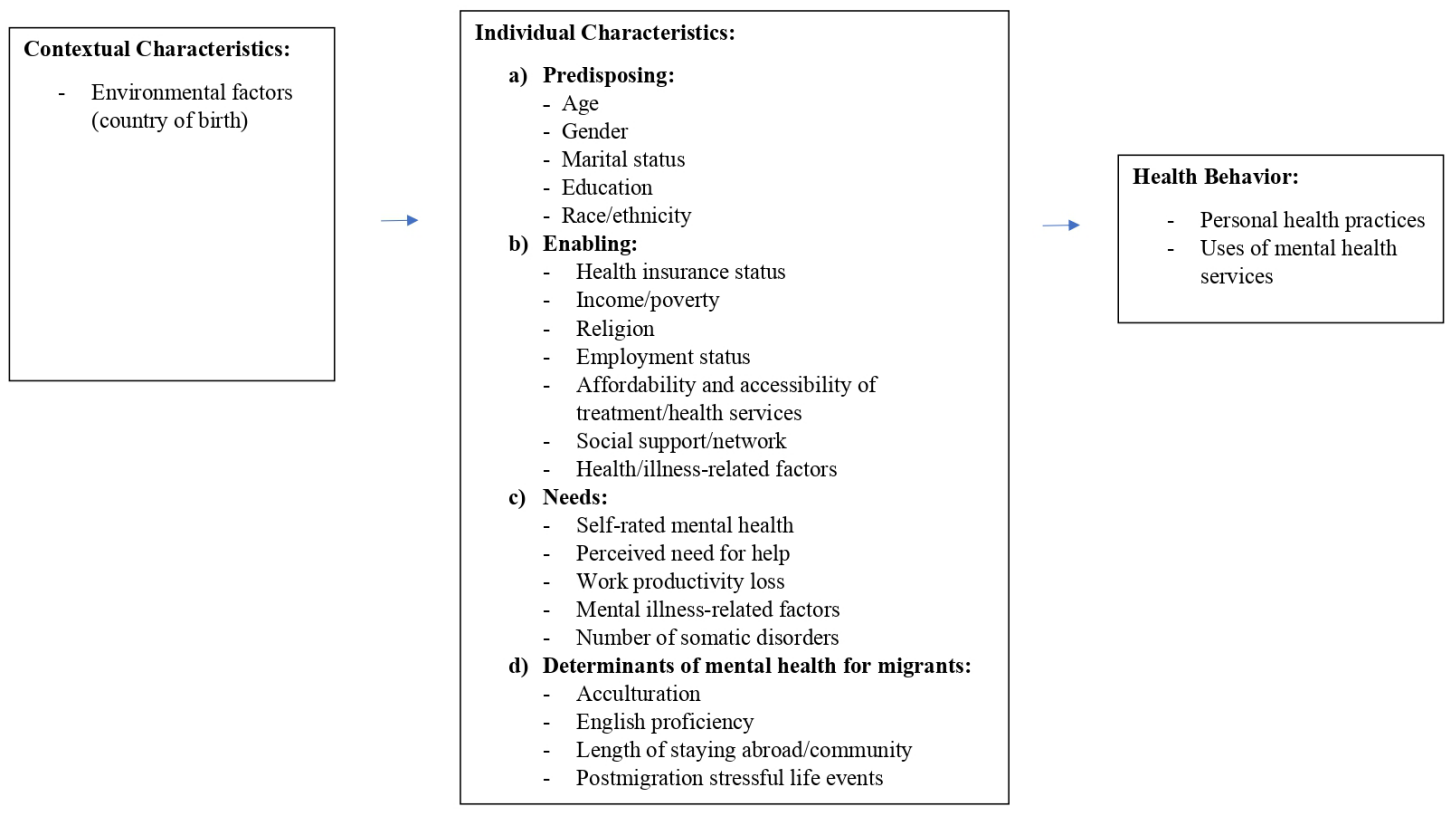
Conceptual model for using in mental healthcare seeking utilization among migrants.

Finally, based on our scoping review, we have constructed a conceptual framework that can be utilized when examining mental healthcare-seeking behavior among migrants (Figure 3). We brought together various elements that played a crucial role in influencing mental healthcare seeking across the 12 studies we examined in our review. Due to the limited number of studies (12) in our review, we incorporated a factor into the framework, even if only a single study demonstrated its significance. Moreover, we distinguished determinants of mental health for migrants as a separate group. In our selected studies, these factors were examined as predisposing, enabling, or separately. We believe that treating them as a distinct group provides clarity for readers, making this group of factors equally important as the other three and easier to understand. We shortly explain our factors selection below.

### Contextual characteristics

Although our review only included one study directly addressing contextual characteristics (45), this aspect is essential when studying the utilization of mental healthcare services. Our scoping review suggests that researchers should consider country of birth, which could be presented by nationality (45).

### Individual characteristics

#### Predisposing factors

In terms of predisposing characteristics, we propose adhering to general recommendations and exploring factors such as age (36, 38–40, 42, 45, 46), gender (35, 36, 39–42, 45), marital status (42, 45), and race/ethnicity (42). We also decided to include education in this group, based on our findings, where two studies identified its significance as a predisposing factor (36, 44), as opposed to one that classified it as an enabling factor (35). Those fundamental factors are suggested based on the results of our scoping review and are also recommended by Andersen (26).

#### Enabling factors

Based on our scoping review, we suggest considering factors such as health insurance status (42), income that includes individual and household income (43), poverty (42), and employment status (35, 37). Additionally, it is important to assess the accessibility and affordability of hospitals and clinics that offer mental health services (38). An interesting factor is religion, which in our studies was found to be a significant factor when seen as one of the enabling factors (35, 46). Additionally, based on the findings from our scoping review, we suggest that researchers should explore the influence of social networks, including family, friends, and community groups, and contact with relatives on mental healthcare seeking (40, 42) as it can be seen as a significant factor that might decrease (40, 42) mental healthcare seeking. Furthermore, we suggest including health/illness-related factors in this group as the two significant factors of health changes over the past year that also includes quality of life (36) were more commonly associated with a general enabling factor than one specific to migrants (Figure 3).

#### Needs factors

According to our scoping review, we recommend considering mental illness-related factors and their severity as a primary need factor in addressing needs (35–44). Additionally, we suggest incorporating self-rated mental health (39, 44), perceived need for help (36, 43), work productivity loss (46), and number of somatic disorders (37) into the assessment of mental health needs.

#### Determinants of mental health for migrants

In this group of factors, we propose considering aspects such as acculturation (35, 41, 46), English proficiency (36, 40, 42), and the length of stay abroad or in the community (36, 43). Moreover, we also believe that it would be beneficial to consider postmigration stressful life events (37) as one of the determinants of mental health of migrants. Combined with other migrant-specific factors, this consideration could allow for a more holistic approach to addressing the health needs of migrant populations.

### Health behavior variables

Our scoping review suggests considering personal health practices and uses of mental health services. Personal health practices should encompass complementary and alternative therapies, self-care coping strategies, and the use of medication for mental health illnesses.

Overall, the Andersen Behavioral Model is a common and powerful theory that can help to discover healthcare services utilization. This model is relatively flexible and adaptable to various populations, including migrants. We presented multidisciplinary findings that can be applied and used by researchers, healthcare providers, and policymakers. However, it is worth remembering that our scoping review identified only 12 suitable studies. Although we made efforts to minimize missing articles by conducting thorough searches across multiple databases and using comprehensive keywords and synonyms, it is still possible that some relevant articles were overlooked (24). Additionally, our study design did not involve a formal critical appraisal of study quality, which is a limitation inherent to scoping studies. Instead of assessing the quality of individual studies, scoping studies aim to provide a descriptive narrative of the literature (23, 24).

Despite the aforementioned limitations, we believe that our study has valuable contributions to the field of mental healthcare, particularly in the context of migrants. By shedding light on the patterns and behaviors of migrants in this area, our research can enhance the understanding of this population and their specific needs. Furthermore, our proposed conceptual framework has the potential to improve outcomes in future studies focused on mental healthcare service utilization among migrants. By considering the unique factors and dynamics that influence this population, our suggested model can enhance the understanding of and provide insights into more effective interventions and strategies.

## Implications

We have proposed implications for researchers, policymakers, and healthcare providers below:

- Researchers—we emphasize the need for more comprehensive research that explores this topic in diverse countries, especially through a qualitative or mixed-methods approach. This recommendation seeks to better understand the perspectives of participants and achieve more reliable results. Researchers should aim to balance the strengths and weaknesses of both quantitative and qualitative study designs. Moreover, we recommend that researchers should consider combining multiple existing versions of the Andersen Behavioral Model to better understand this complex topic. We emphasize the need for more studies that explore health outcomes and contextual factors specific to migrants, such as health policy, costs, availability of mental healthcare services, and the ethnic and racial composition of the population. We also create a conceptual framework that can help researchers to conduct research on migrant populations.
- Policymakers—we suggest that healthcare policies for migrant populations may differ from those for native citizens, and these differences can also vary among immigrants from different countries. Policymakers should take these variations into account when designing healthcare policies for migrants.
- Healthcare providers—to provide effective mental healthcare to migrants, healthcare providers should consider their definition of mental healthcare in a broader context, including health policies, costs, availability of mental healthcare services, occupation changes, legal status (including legal and illegal migrants, asylum seekers, and refugees), acculturation, language ability, and generational status. Healthcare providers should work to create culturally competent and stigma-free environments for mental healthcare services.

## Conclusion

In summary, our scoping review highlights the need for further research using the Andersen Behavioral Model to investigate mental healthcare service utilization among migrants. Firstly, it is noteworthy that some researchers combined different versions of the model to adapt it to the unique characteristics of migrant populations. This approach can offer valuable insights into how the model can be tailored to specific contexts. Secondly, the review reveals that individual characteristics, particularly predisposing factors, were widely studied, emphasizing their importance in understanding healthcare utilization among migrants. Thirdly, the dominance of quantitative approaches in the selected studies emphasizes the need for more qualitative or mixed-methods approaches. Finally, we have proposed a conceptual model based on our review that can be used to study mental healthcare-seeking behavior among migrants and better organize and analyze complex data, clarify relationships between variables, and inform policy development.

## Data availability statement

Any data from the ongoing research can be obtained from the H-RL upon request.

## Author contributions

EK: Conceptualization, Data curation, Formal analysis, Methodology, Project administration, Writing – original draft, Writing – review & editing. OA: Conceptualization, Visualization, Writing – review & editing. H-RL: Conceptualization, Formal analysis, Methodology, Project administration, Supervision, Writing – review & editing.
